# Indents

**Published:** 1913-05-15

**Authors:** 


### INDENTS.

☞Brief Articles of Technical or Scientific Value will receive notice and comment. Contributions invited.

*Matrices.* Place a strip of annealed copper, of correct width, around the tooth, pinch the same together until reasonably close-fitting at the greatest circumference of the tooth, remove, touch the same with zinc chlorid solution and solder in the Bunsen flame with tinner's solder. This band, whose walls are now practically parallel, is replaced upon the tooth, wedged at the gingival portion, and then pinched tightly against the other portions of the tooth. This makes a matrix that supplies absolutely a missing wall, the only fault being that it will, as stated before, become distorted if too great force is applied during the filling process.

A matrix that is but infrequently used—for the reason, I think, that but few have heard of it, or, if having heard of, have failed to try it—is one suggested, I believe by the late Dr. Bonwill. It is of the greatest value in building up with amalgam those molar teeth where a considerable portion of the buccal or ligual wall is missing. It is made by simply pressing against the cavity wall and adjoining teeth a small piece of softened modeling compound. This is now chilled, removed, and with a keen-edged penknife the compound is cut away at that portion representing the missing tooth wall. This matrix may be in position by the fingers or ligated to the adjoining teeth, during insertion of the filling.

A method of overcoming a difficulty often experienced in adapting a matrix to an approximo-occlusal cavity when the cervical portion of the missing wall presents a concave surface due to the bifurcation of the roots may be made by the use of sheet copper 5-1000 of an inch in thickness, in conjunction with a mechanical matrix and holder, or an additional strip of copper or steel held by wedges or ligatures an appliance may be quickly made which, when removed after the filling has been inserted, will be found to have kept the filling the desired shape, leaving no overhanging portions at the gingival margin to trim away.

A piece of the copper plate, thoroughly annealed, and large enough to extend, say, one-eighth of an inch beyond the buccal and lingual margins, is pressed with cotton or bibulous paper pellets to conform to the concave root periphery at the cervix.

The copper is then carefully removed, the concave portion of the plate is touched with zinc chlorid, and over an alcohol or small Bunsen flame soft solder is flowed into the depression, any excess solder being removed with a disk.

The prepared copper plate is then replaced in position, around it the mechanical or other matrix is adjusted, and when fully tightened the free edge of the copper is burnished against the steel.

If the cavity is to be filled with amalgam, hard wax instead of solder may be used for filling up the concave surface of the copper. In either case a slight excess of contouring material is of advantage, in that the copper plate is made to hug the tooth more tightly.—Dr. A. P. Lee in *Brief.*

*Bromural in Dental Practice.* Bromural is a combination of bromine, valerianic acid and urea. It is put up in form of tablets, each containing 5 grains of Bromural and 3 grains of milk sugar. They have a distinct odor of valerian, and crumble quickly in water, but they do not dissolve. They are given internally in water. The taste is slightly bitter. The dose for children up to the age of 12 is 5 grains or 1 tablet; for older children or adults 10 grains or two tablets. There is no object in giving a larger dose.

About 20 or 30 minutes after administration the tension of anxiety disappears. The sense of pain still remains, but the patients become more indifferent to it. They permit the necessary needle puncture, for local anesthesia, to be made quietly, or they make ineffectual resistance against it. In the same way they allow the subsequent treatment to be performed.

It is well known that not much can be done with children under local anesthesia, but Bromural has yielded the very best service in children. Refractory children who do not allow themselves to be examined are so far calmed by one tablet, that the diagnosis can be made and even the injection be performed with a good result. For example, I was able to operate under terminal anesthesia, in Dec. 1911, on a cyst of the lower jaw, as large as a hen's egg, extending to the ascending ramus, in the case of a boy aged 11 years, after giving him two tablets (exceptional). The operation lasted 20 minutes, and the child did not manifest any sign of pain. He lay the whole time, in semi-sleep, with his eyes closed. Five days later I was fortunate enough to effect the same in a parallel case of left-sided cyst of the lower jaw, in a girl aged 8 years. I bring these cases forward, because they involved rather serious and prolonged operations at least, for children—cases in which I would probably have resorted to general anesthesia previously.

In several cases I have not only given Bromural to the children, but also to their mothers who accompany them, for they often unnecessarily increase the excitement of their apprehensive children by their own foolish attitude. After Bromural, both mother and child become calm.—Professor Williger, Berlin, in *Deutsche Zahnaerztliche Zeitung*, 1912, No. 12.

*To Make Ideal Gold Dummies.* After procuring the model bearing the attachments, select from your reserve stock of porcelain teeth those which approximately fit the case, imbed them in plaster, tip them out and anoint the mole thus obtained with glycerine, burnish in casting wax, remove and make any corrections necessary by shaving the wax. Cast and you have an ideal piece of work.—C. E. Allen, Chicago.

*Etching on Steel.* Cover the article with a film of paraffin wax, and with a scriber write or mark whatever is required. Sprinkle some salt over this, and then cover with strong nitric acid. Clean off with hot water, and grease article to prevent rusting.

*Value of a Complete Diagnosis.* So many people get the idea that a certain tooth is giving them trouble, yet it needs only a glance to see that the whole mouth is in trouble. I have noticed this especially among the younger men; they either do not recognize the conditions, or they are opposed to telling the patient that the whole mouth is involved instead of a certain few teeth. The failure to grasp the condition is a serious thing. Simply treating the one or two teeth that the patient wants to have treated is a mistake. Make models and study these things as othodontists would, and make up your mind where the trouble is and what is to be done, and you will find, as in many of these larger cases that I passed around, instead of one part of the mouth being treated, sometimes, as the patient intended, they go away with the whole of the mouth changed. It is the duty of the dentist to correct the conditions from the proper standpoint.—Dr. W. D. N. Moore in *Items*.

*Drills, Reamers and Broaches.* The smaller sizes of these useful tools are frequently required in the dentist's workroom, especially since the advent of modern orthodontia appliances. The simplest form of drills are those known as "spear head." They are easily made and easily kept in order, and for some purposes are more desirable than the twist and fluted drills which have in recent years displaced them. Spear head drills are difficult to keep to exact sizes, and as usually made are scraping tools, and not as effective on noncrystalline metals as the modern twist drills which are cutting tools. Twist and fluted drills are commercially made in exact standard sizes, and with care in sharpening can be depended upon to closely maintain their size until worn out. Twist drills are not suitable for thin metal; when given sufficient pressure to cut they have a tendency to "draw in", making a figure-of-eight-shaped hole, and to either break the drill or tear the metal, or if the workman is not watchful may cause the metal to lacerate his fingers. The fluted drill is an effective scraping tool, and is especially useful on such metals as brass. It is also useful for enlarging holes, as when so used it does not draw in, as does a twist drill. No drill can be depended upon to make a hole perfectly round or to exact size; where either exactness in size or shape is required the drill must be followed with the reamer. A properly made reamer is an exact tool; it leaves the hole straight and exact as to shape and size, and that is its principal use. They are usually fluted, more or less tapered, and may be sharpened with but little, if any, change in size. A broach is used to enlarge a hole where exact size or shape is not important. They are tapered but not fluted, usually four or five sided (the five-sided works much smoother and leaves the hole more nearly round), and are especially useful for enlarging holes in thin metal.—W. H. T. in *Brief.*

*Solder Bench.* Dr. E. Carlton Palmer, Philadelphia, has on his work-bench a handy arrangement in which he does all his soldering and in which he keeps all his soldering tools and appliances. It consists of a compartment about one foot square, the bottom, back and two sides made of half-inch asbestos board. (Asbestos board is a commercial article, not at all expensive, and fireproof.) Within this he has the support for the work he is soldering, and close at hand an arrangement for heating up the work preparatory for soldering, an ordinary and a fine-flame blowpipe, both supplied with compressed air, and the various tools, flux, etc., use in soldering operations. The sides of the compartment protect from air currents that now and again prove a distraction, and are a protection against fire.

He has all that he needs close at hand, each in its appropriate place. As a solder support when soldering porcelain teeth he uses a fireclay box, round, and of a size and depth to accommodate an invested full denture. The cover is cut from the bottom part on a diagonal line, so that the side does not interfere with the free use of the blowpipe. When the soldering is complete the cover is placed in position, converting it into an annealing oven. The rubber tubes conveying gas and air come up from underneath the bench, so that in working they are never in the way, nor in danger from the gas flame.—*Brief.*

*Home-Made Tools and Appliances.* At the present time the manufacturer of tools and appliances of all kinds has reached a point of quality and cheapness, and the market is so well supplied that make-shifts of home manufacture have, as a rule, lost their one-time value. Nevertheless, the ability to quickly improvise a tool or appliance to meet an emergency is well worth cultivating. While much depends upon one's mechanical skill, workroom conveniences and inventive ability, "home-made," if the making consumes time that otherwise would be occupied in money earning, is not true economy. If used as a pastime, or if one's time is not fully occupied, and the results are satisfactory and answer the purpose fully as well as a commercial article, then the home-made product is a money saver. The expert mechanic in constant daily practice, whose work, aided by approved machinery, has been reduced to mere routine, can turn out results that are beyond the reach of even an unusually skilful amateur; and at a cost to the user but little more than the retail price of the materials employed. It is quite impressive to see at a clinic a wobbly carborundum dental engine wheel in a few minutes made to run "true as a die" and to cut as well as it ever did; but does it pay to stop in the midst of an operation to accomplish this when a new wheel costs but a few cents? There are exceptions, of course, but before spending time and cash in getting up some recommended home-made contrivance that may work "after a fashion" it will be wisdom, to see what is the difference in cost between it and an approved, well-made device, tried and tested and ready for business.—W. H. T. in *Brief*.

*Simple Means for Polishing Fillings and Cleaning Teeth.* Around an old inverted-cone bur, while it is revolving in the engine, cotton is wound in sufficient thickness so that the bur will not cut through the cotton. This polisher is dipped in a solution of pumice and water for polishing amalgam fillings and cleaning the teeth. For polishing gold inlays and fillings, it can be dipped in prepared chalk and water.—F. Schwartz, *Dental Review*.

*Technique of Making an Accurate Matrix for Porcelain Inlays.* The following is a description of the technique of using medium weight China silk, instead of goldbeaters' skin, as suggested by Dr. Emil Schreier of Vienna, as a cradle support for gold or platinum in obtaining matrices for inlays. The silk may be regarded as an improvement over the velum, as it has greater bulk and better sustains the foil.

The foil being cut sufficiently large to cover the prepared cavity amply, is placed between a fold of silk, dipped in water, and with a wet pellet of cotton or spunk is quickly carried to the floor of the cavity. With a suitable instrument in one hand it is held firmly in position; in the other hand, with ball-pointed pliers, the metal is grasped, together with the silk, pulling and smoothing out all folds or wrinkles. Then it is packed full with wet cotton or spunk, using heavy pressure, turning all margins with a large pellet of wet spunk held in the ball-pointed pliers. It is then taken from the cavity, the silk is carefully removed, and the matrix returned for completion by again swaging with pellets of cotton or spunk, this time used dry. The operation is completed by filling with wax or gum camphor for safe removal. At the same time all margins are made perfect with a roller burnisher. The matrix is then removed and invested. When the wax has been melted or boiled out, the porcelain is introduced and baked in the usual manner. Much time can be saved, however, with a smaller number of bakings, by packing the wet porcelain into the matrix with a short, stiff camel's-hair brush, absorbing the moisture after the introduction of each layer of porcelain.—E. S. Gaylord, *Dental Brief.*

*A Case of Death under Nitrous Oxid and Oxygen.* The patient, a very stout man of fifty-three, exhibiting unmistakable symptoms of emphysema and coronary sclerosis, was given 0.01 cc. of morphin hypodermically half an hour before the operation. As usual, a high percentage of nitrous oxid was administered at the beginning of the anesthesia, followed by a gradually increasing percentage of oxygen. The patient became suddenly cyanosed, and, although pure oxygen was at once turned on, ceased to breathe; a slight twitching of his legs was observed. After another thirty seconds, pronounced pallor ensued, despite artificial respiration and ether hypodermically, and the heart-beat could no longer be perceived. The air could not be made to pass properly through the nose and mouth although the tongue was held well forward, and no obstacle to respiration could be detected. The post-mortem showed luetic aortitis, secondary hypertrophy of the heart, sclerosis of the coronary arteries, and degeneration of the heart muscle.

The author attributes the asphyxia to the narrowing of the oro-pharyngeal aperture ensuing upon the light cyanosis occurring during nitrous oxid anesthesia, and giving rise to impeded respiration, especially through the mouth, which as Threwby has pointed out, occurs mostly in cases of large and flabby tongue.

In the case described, the short asphyxia sufficed to produce symptoms of heart collapse. In similar cases, a quickly instituted tracheotomy might save the patient's life.—Dr. John Olow, *Cosmos.*

*Removing Deposits from German Silver Appliances.* Regulating appliances of German silver can be freed of deposits and discoloration by ordinary pure hydrochloric acid applied cold. The cleaned appliances are immersed in a solution of sodium bicarbonate.—*Archiv fuer Zahnheilkunde.*

